# Circadian rhythmicity in murine blood: Electrical effects of malaria infection and anemia

**DOI:** 10.3389/fbioe.2022.994487

**Published:** 2022-11-10

**Authors:** Fatima H. Labeed, Andrew D. Beale, Petra Schneider, Stephen J. Kitcatt, Emily J. Kruchek, Sarah E. Reece

**Affiliations:** ^1^ Centre for Biomedical Engineering, University of Surrey, Guildford, United Kingdom; ^2^ Institute of Ecology and Evolution, University of Edinburgh, Edinburgh, United Kingdom

**Keywords:** dielectrophoresis, malaria, malaria-induced anemia, electrophysiology, DEP, mouse, parasitemia, rhythm

## Abstract

Circadian rhythms are biological adaptations to the day-night cycle, whereby cells adapt to changes in the external environment or internal physiology according to the time of day. Whilst many cellular clock mechanisms involve gene expression feedback mechanisms, clocks operate even where gene expression is absent. For example, red blood cells (RBCs) do not have capacity for gene expression, and instead possess an electrophysiological oscillator where cytosolic potassium plays a key role in timekeeping. We examined murine blood under normal conditions as well as in two perturbed states, malaria infection and induced anemia, to assess changes in baseline cellular electrophysiology and its implications for the electrophysiological oscillator. Blood samples were analyzed at 4-h intervals over 2 days by dielectrophoresis, and microscopic determination of parasitemia. We found that cytoplasmic conductivity (indicating the concentration of free ions in the cytoplasm and related to the membrane potential) exhibited circadian rhythmic behavior in all three cases (control, malaria and anemia). Compared to control samples, cytoplasm conductivity was decreased in the anemia group, whilst malaria-infected samples were in antiphase to control. Furthermore, we identified rhythmic behavior in membrane capacitance of malaria infected cells that was not replicated in the other samples. Finally, we reveal the historically famous rhythmicity of malaria parasite replication is in phase with cytoplasm conductivity. Our findings suggest the electrophysiological oscillator can impact on malaria parasite replication and/or is vulnerable to perturbation by rhythmic parasite activities.

## Introduction

Circadian rhythms are widespread biological adaptations to the day-night cycle, whereby organisms physically alter to adapt to changes in their environment or internal physiology according to the time of day, and such rhythms can be found in the majority of cells in the body. A number of mechanisms for the regulation of cellular circadian clocks have been identified. For example, red blood cells (RBCs) contain an oscillator (O’Neill and Reddy, 2011) which operates without transcriptional control. Whilst the mechanism of the RBC oscillator is yet to be fully elucidated, it does contain an element related to cytosolic potassium (K^+^), which cycles in concentration across a 24-h period. Since K^+^ is fundamental to membrane potential (which exhibits circadian behavior in the same cells) as well as cytoplasm conductivity (the ability of the cytoplasm to conduct an electric current), K^+^ cycles are consistent with an electrophysiological rhythm (Henslee et al., 2017).

Understanding the drivers and consequences of cellular rhythms is challenging because conventional tools for electrophysiological measurement are not amenable to high-time-resolution study of electrical rhythms, due to their speed and complexity. For example, patch clamp can typically measure no more than a few cells per day. An alternative approach uses a phenomenon called dielectrophoresis (DEP). DEP is a rapid, label-free and non-invasive technique for determining mean measures of parameters such as the electrical capacitance of the membrane, and the electrical conductivity of the cytoplasm of cells. Typically 20,000 cells are measured simultaneously, with the measurement process taking less than a minute (Graham et al., 2015; Hoettges et al., 2019). DEP has revealed circadian cycles in the electrical properties of RBCs (Henslee et al., 2017; Beale et al., 2019; Beale et al., 2022) human as well as sub-2 h, ultradian rhythms in the RBCs of voles (Hoettges et al., 2019). More recently, connections have been demonstrated between these DEP-derived electrical parameters and other electrical measures of cell function, such as the membrane potential (V_m_) and the extracellular electrical potential (the ζ-potential) that regulates the way cells interact with other cells and biological entities in their immediate environment (Hughes et al., 2021; Hughes et al., 2022).

Whilst the rhythmic behavior of healthy RBC electrophysiology has been well-characterized, whether RBC rhythms are altered by blood diseases remains unknown. To address this, we compared the electrophysiological rhythms of healthy (control) murine blood samples to those affected by one of two blood states; malaria infection and anemia. To induce anemia, we treated mice using Phenylhydrazine (PHZ) which destroys RBC, stimulating erythropoiesis and generating a reticulocyte rich RBC population, as well as altering RBC physiology by increasing glucose consumption, ATP depletion, and lipid peroxidation (Magnani et al., 1988). To induce malaria, mice were infected with parasites of the rodent model, *Plasmodium chabaudi*. *Plasmodium* is transmitted between hosts *via* an insect vector and in the initial infection stage, replicates asexually in the liver. Following this, the parasites enter an erythrocytic phase and undertake sequential, synchronized, cycles of asexual replication within RBCs (Prior et al., 2020). Asexual replication during the erythrocytic phase of malaria infection is famously rhythmic, causing fever when infected RBC burst at intervals lasting 24, 48 or 72 h depending on the species (Garcia et al., 2001). Asexual replication also fuels the production of transmission forms and is responsible for the severe symptoms of malaria infections, so a better understanding of rhythms in parasite replication might reveal novel targets for malaria treatments.

Malaria-infected RBCs exhibit changes to the RBC membrane and cytoplasm. The electrical properties of RBCs before and after infection with *Plasmodium falciparum* are a function of medium conductivity (Gascoyne et al., 1997; Panklang et al., 2022), and membrane conductivity is associated with changes to membrane potential (Rijo-Ferreira et al., 2020). Furthermore, quinine, a long used antimalarial drug, is a K^+^-channel blocker and alters the electrical conductivity of the cytoplasm (Duncan et al., 2007). Additionally, human reticulocytes, associated with anemia, have a lower mean membrane potential than RBCs (Moura, 2020). Whether this alteration of cellular electrical properties is due to circadian activity and/or changes in the age structure of the RBC population are unknown. For example, whether the difference in membrane potential between uninfected and malaria-infected RBCs is explained by changes across the plasma membrane of the host cell, or across membranes of both the host and the parasite, or differences in the electrical potential at the hydraulic plane of shear (the plane surrounding the cell, inside of which the water molecules and ions are bound to the cell surface), the ζ-potential, are not known. Given the multiple roles K^+^ plays in both the generation of membrane potential and as a component of the electrophysiological RBC clock, we hypothesized that there may be an electrical component to both infectivity and rhythmicity in malaria parasite replication and RBC rhythms.

To separate the impact of malaria infection on RBC electrophysiology from that simply due to the effects of anaemia, we compared the electrophysiological rhythms of RBCs from mice infected with *Plasmodium chabaudi* to control RBC from healthy mice and from mice following PHZ treatment. To assess circadian rhythmicity in all RBCs, we analyzed the DEP spectra of murine erythrocytes at 4 h intervals, covering a 48 h period for all three groups. Measurements were taken of cytoplasm conductivity, membrane capacitance, parasitemia and RBC density. Results indicate that both anemia and malaria infection affect RBC rhymthms in different ways.

## Materials and methods

### Experimental design

**FIGURE 1:**
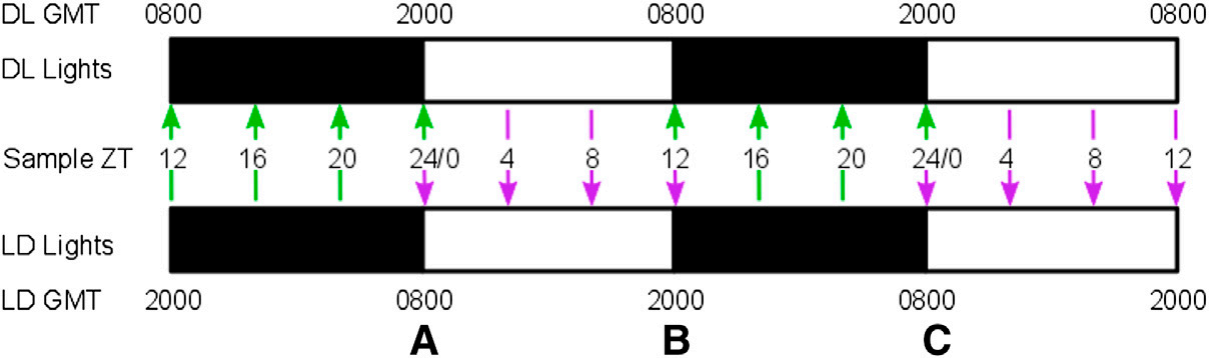
Sampling regime. Mice were allocated to one of two 12 h dark: 12 h light lighting regimes, with lights on at 0800 GMT for the standard lighting regime (LD) and 2000 GMT for the reversed lighting regime (DL). Black and white bars indicate time of dark and light respectively. The offset lighting regimes were used to obtain 4-hourly sampling across a 48 h timeframe by concatenating samples from the DL group (green arrows) and LD group (purple arrows), as indicated by the sampling day and ZT time (Zeitgeber time, i.e., hours since lights on). This design generated three points of overlap **(A–C)** when both LD and DL mice were sampled at comparable points to verify each cohort within each treatment group is a good replicate of the other. RBC densities were collected for both cohorts at the second and last overlaps, and parasitemia was measured for all three overlaps.

Hosts were C57BL/6J female mice, 9–10 weeks old, housed at 21°C, with unrestricted access to a standard RM3 pelleted diet (801700, SDS, United Kingdom) and drinking water supplemented with 0.05% para-aminobenzoic acid. 30 mice were assigned to two lighting regimes (Figure 1, lights on 20:00–08:00 GMT (DL) and lights on 08:00–20:00 GMT (LD)), 15 in each regime. All mice were allowed 2 weeks to acclimatize (“entrain”) to their respective light-dark rhythms before the experiment began. Mice within each lighting regime were assigned to three different treatments: malaria infection (Pch), PHZ (PHZ) treatment, or untreated controls, with five mice per treatment. PHZ treatment was administered by intraperitoneal injection at lights on (Zeitgeber Time 0, ZT0) at a dose of 120 mg/kg, 5 days (LD) and 4 days and 12 h (DL) days before the start of sampling (Birget et al., 2017). Malaria infections were initiated 4 days (LD) and 3 days and 12 h (DL) days before the start of sampling by intravenous injection of 1 × 10^7^ *P. chabaudi* (genotype AJ) infected RBCs originating from donor mice kept in the same lighting regimes as the experimental mice (O'Donnell et al., 2011; Prior et al., 2019). PHZ and parasite injections were administered at 08:00 (LD) and 20:00 (DL). All procedures were carried out in accordance with the United Kingdom Home Office regulations (Animals Scientific Procedures Act 1986; SI 2012/3039) and approved by the ethical review panel at the University of Edinburgh. All mice were sampled at 08:00, 12:00, 16:00 and 20:00 GMT for 2 consecutive days, thus covering 4-hourly sampling over a period of 48h, across LD and DL cohorts for each treatment group (Figure 1). The use of LD and DL cohorts enabled both halves of the circadian cycle to be simultaneously investigated whilst minimizing the amount of blood withdrawn for sampling to avoid sampling itself confounding our experimental perturbations. Furthermore, sampling both cohorts with an overlap between groups at handover points (Figure 1) enabled us to ensure the LD and DL cohorts were sufficiently similar to each other to allow their time series to be concatenated (O’Donnell et al., 2022). Thus, five mice were measured at each timepoint, with ten mice contributing to each of the overlaps.

### Data collection

At each sampling point, a thin blood smear was prepared and stained with 10% Giemsa to quantify the proportion of infected RBCs for malaria infected mice. Every 12 h, at the overlaps for LD and DL cohorts, RBC densities per ml were measured by flow cytometry (Z2 Coulter Counter, Beckman Coulter) immediately after sample collection. At each sampling time, 10 µl of blood was collected from the tail vein, and suspended in 5 ml of DEP buffer (consisting of 248 mM sucrose, 16.7 mM dextrose, 250 mM MgCl_2_, 100 mM CaCl_2_, 290–299 mOsm, altered to a conductivity of 0.017 S/m by adding DPBS (Sigma Aldrich, United Kingdom)). Electrophysiological properties were measured using the 3DEP 3D Dielectrophoresis Cell Analysis System (3DEP reader, Labtech, United Kingdom). The cell suspensions were injected into 3DEP chips (Labtech, United Kingdom) and read on the 3DEP reader (Labtech, United Kingdom). The wells in the 3DEP chip were collectively energized for 30 s at 10 Vp-p at 20 different frequencies between 10 kHz—20 MHz. At 12:00 and 16:00 GMT, a 10 µl aliquot of each sample was placed into a C-Chip hemocytometer (Labtech, United Kingdom) and pictures were taken on an Olympus BX43, with a digital camera (Microtec MDC-5C). The images were used to measure the diameter of around 100 cells using ImageJ, version 2. The average radius was calculated prior to DEP modelling. Raw data was fitted using the single-shell model (Broche et al., 2005) in MATLAB to extract the electrophysiological parameters of cytoplasmic conductivity, σ_cyto_ and membrane capacitance C_eff_. Note that the use of the single-shell model considers the cytoplasm as homogeneous and does not therefore model the parasite-infected cells separately; this is to ensure a unique solution can be determined when fitting to a DEP spectrum with three identifiable variables - the initial state, and the frequencies at which the spectrum rises and falls. Using an additional internal shell to represent the parasite would add two additional parameters and prevent the identification of a unique solution.

### Data analysis

**FIGURE 2:**
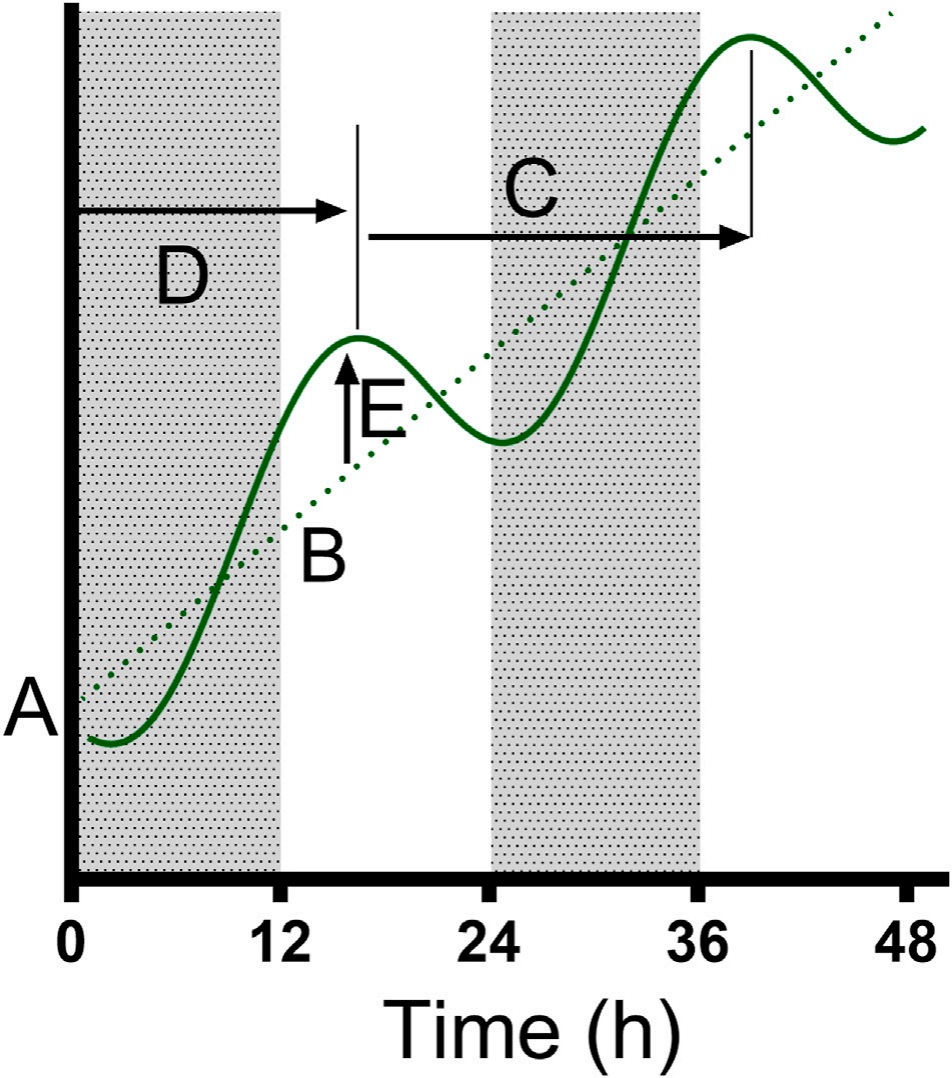
The parameters fitted to the data. We used a model where a cosinor (solid curve) oscillates around a line (dotted line); the model was classified by the initial value **(A)** and gradient **(B)** of the line y = Bx + A, as well as the period **(C)**, Acrophase (time to first peak, **(D)** and amplitude **(E)** of the cosinor. Where a cosinor was not preferred to a straight line, only the preferred parameters of the straight line were recorded. The model was able to identify “damped” cosinors with variable amplitude across the 48 h period.

Data were analyzed in Prism 9 (Graphpad Software, La Jolla, CA) to test the presence of a biological rhythm in measured parameters. First, we compared the RBC densities at the overlap sampling points (ZT24/0, 12; Figure 1) between the LD and DL cohorts for each treatment group, and parasitemias (% parasite-infected RBCs) at the overlaps (ZT24/0, 12; Figure 1) for the Pch group, using an unpaired *t*-test with Welch’s correction. RBC densities did not vary according between LD and DL cohorts for the control group (overlapB: *p* = 0.667; overlapC: *p* = 0.722), nor the PHZ group (overlapB: *p* = 0.909; overlapC: *p* = 0.330), nor the Pch group (overlapB: *p* = 0.877, overlapC: *p* = 0.446). Likewise, within the Pch group, the LD and DL cohorts did not differ in parasitemia for any of the overlaps (overlapA: *p* = 0.0642; overlapB: *p* = 0.635; overlapC: *p* = 0.864). Having revealed no biologically relevant differences in the underlying blood and parasite dynamics between the cohorts, we concatenated the cohorts to produce a 48 h time series to analyze the DEP parameters and parasitemia [Figure 1, (O’Donnell et al., 2022)]. We tested whether each 48-h time series exhibited rhythmicity by comparing whether a damped cosine + baseline model (Figure 2) provides a better fit to the full data set (using individual measurements from each mouse) than a straight line using non-linear regression comparison of fits (H1 = damped cosine model, H0 = straight line) extra sum-of-squares F test; the straight line was preferred unless the *p*-value was <0.05. Where rhythmicity was observed, key characters from the model were extracted: the period, amplitude and phase of the cosinor component, as well as the initial value and gradient of the line (the baseline) around which the cosinor oscillates (or the line which is preferred to a cosinor where *p* > 0.05). Period, phase, amplitude and baseline values were derived from the best fit cosinor, and compared using the extra sum-of-squares F comparison test and 1-way ANOVA (Kruskal–Wallis). If ANOVA revealed significant (*p* < 0.05) effects, Holm-Sidak’s multiple comparison post hoc tests were used to determine *p* values for all relevant comparisons.

## Results

We first report assessments of rhythmicity in the dielectric properties of the cytoplasm conductivity σ_cyto_ and effective membrane capacitance C_eff_ of blood from three types of hosts—uninfected controls, as well as malaria-infected (Pch), and anemic (PHZ) mice. We then probed potential relationships with parasite rhythms. The data are summarized in Table 1.

**TABLE 1:** The results (± standard deviation) of fitting a damped cosinor model to the experimental data. Where the model fits preferentially to a straight line (*p* < 0.05) the period, phase and amplitude are included, as well as the initial value and gradient of the baseline around which the sinusoid oscillates; where the straight line is preferred, the initial value and gradient are provided.

	σ_cyto_			C_eff_			Parasitemia
	Control	Malaria	Anaemia	Control	Malaria	Anaemia	Malaria
Cosinor in preference to straight line?	Yes *p* = 0.017	Yes *p* = 0.022	Yes *p* = 0.039	No *p* = 0.11	Yes *p* = 0.034	No *p* = 0.099	Yes *p* < 0.0001
Value at t = 0	0.147 (±0.003)Sm^−1^	0.148 (±0.003)Sm^−1^	0.140 (±0.002)Sm^−1^	0.013 (±0.001 Fm-^−2^	0.014 (±0.006 F m^-2^	0.016 (±0.001) Fm^−2^	12.4 (±1.7)%
Gradient	-2.2e-5 (±1e-4) Sm^−1^h^−1^	1.6e-5 (±1e-4) Sm^−1^h^−1^	2.3e-5 (±9e-5) Sm^−1^h^−1^	-7.0e-5 (±1.8e-5) Fm^−2^h^−1^	-9.2e-5 (±2e-5) Fm^−2^h^−1^	-0.0001 (1.9e-5) Fm^−2^h^−1^	0.77% (±0.06)h^−1^
Period	25.5 (±1.8) h	25.0 (±2.9)h	23.1 (±0.0)h	-	24.6 (±1.7)h	-	22.5 (±1.2) h
Acrophase	20.2 (4.1)h	11.5 (6.1)h	16.2 (0.1)h	-	19.3 (±3.9)h	-	15.0 (±2.8)h
Amplitude	0.007 (±0.002)Sm^−1^	0.005 (±0.002)Sm^−1^	0.010 (±0.002)Sm^−1^	-	0.002 (±0.000)Fm-2	-	7.32 (±1.1)%

### Cytoplasm conductivity (σ_cyto_)

**FIGURE 3:**
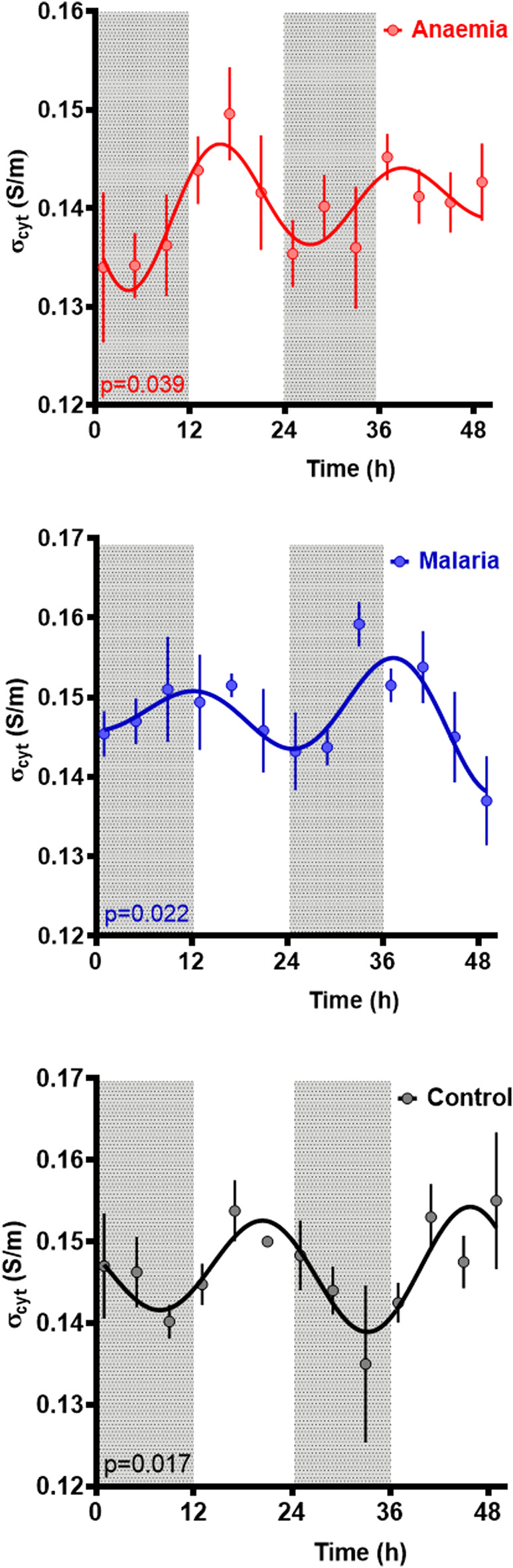
Cytoplasm conductivity for RBCs from untreated controls, malaria-infected and anaemic mice. Mean ± SEM and best fitting models for the cytoplasmic conductivity σ_cyto_ as a function of time since the experiment began, with ZT indicated by light and dark panels. Color-matched curves and lines represent best-fit models; cosinors are used where more statistically appropriate (*p* < 0.05) than straight lines. Colors represent measurements from RBC from untreated (black), malaria-infected (blue) or anemic hosts (red).

Rhythmic patterns of σ_cyto_ were observed in control (*p* = 0.018), PHZ treated (*p* = 0.039), and malaria-infected (Pch, *p* = 0.022) mice (*p* values reflect cosinor fits relative to a straight line), suggesting rhythmicity in all the conditions (Figure 3). Period estimates from control mice (25.5 ± 1.8 h) and malaria infected mice (25.0 ± 2.9 h) were similar, whereas RBC from anemic mice exhibited a shorter period (23.1 ± 0.0 h); however, these differences were not significant (Welch’s ANOVA test, F_(2, 143.7)_ = 0.31, *p* = 0.73). Collective statistical analysis of all three groups suggested a trend in baseline σ_cyto_ to be lower in PHZ relative to other groups, though these were not significant (Welch’s ANOVA test, F_(2.00, 148.6)_ = 2.63, *p* = 0.098). A difference in acrophase was noted, with control cells peaking 20.2 ± 4.1 h after the start of observations, malarial cells peaking at a much shorter 11.5 ± 6.1 h, and anemic cells at an intermediate 16.1 ± 0.1 h.

### Membrane capacitance (C_eff_)

**FIGURE 4:**
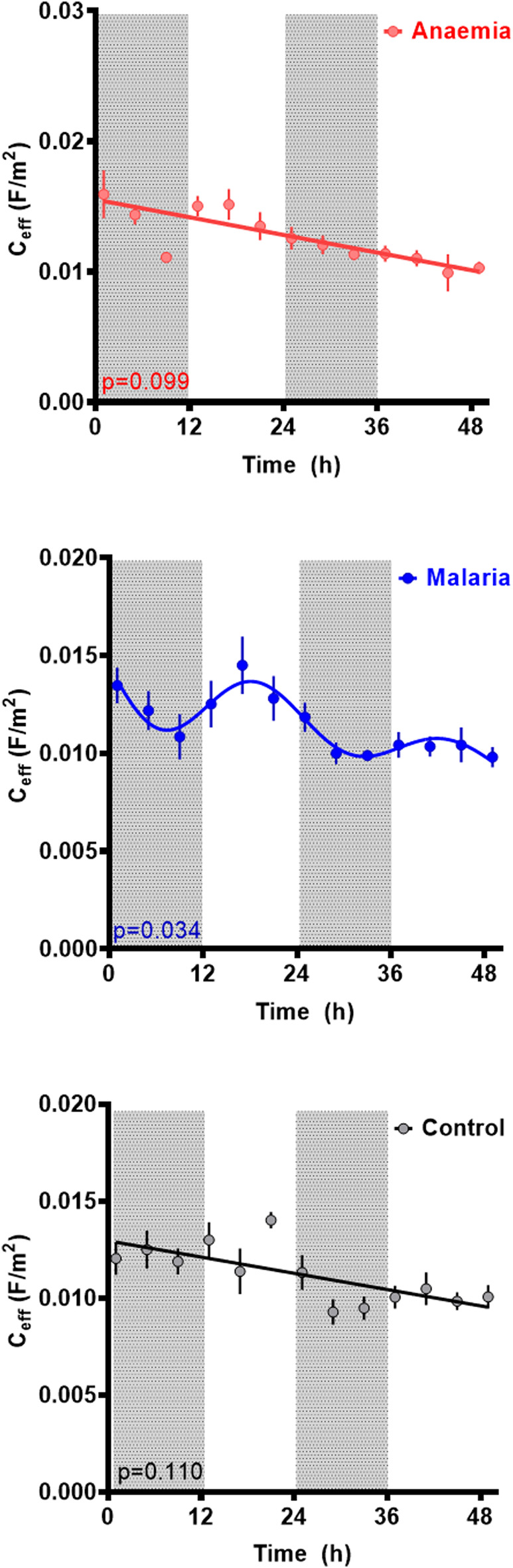
Membrane capacitance (C_eff_) per unit area for RBCs from untreated controls (black), malaria-infected (blue) and anemic mice (red). Mean ± SEM and best fitting models for C_eff_ as a function of time since the experiment began, with ZT indicated by light and dark panels. Color-matched curves and lines represent best-fit models; cosinors are used where more statistically appropriate (*p* < 0.05) than straight lines. Colors represent measurements from RBC from untreated (black), malaria-infected (blue) or anemic hosts (red).

The membrane capacitance across the three conditions can be seen in Figure 4. A statistically significant rhythm (*p* = 0.043, cosinor fitted relative to a straight line) was observed in C_eff_ in the Pch group, with a period of 24.6 ± 1.7 h, an acrophase of 19.3 ± 3.9 h, and an amplitude of 0.002 ± 0 Fm^−2^. Collective statistical analysis of all three groups revealed baseline C_eff_ to be significantly different lower for anaemic mice relative to other groups (two-way ANOVA, F _(2,197)_ = 8.038, *p* = 0.0004; vs. control *p* = 0.0006, vs. Pch *p* = 0.0078). Whereas C_eff_ for malaria infected mice was not statistically different to control mice (*p* = 0.73). A downward trend in baseline C_eff_ values over time was observed in all three groups, though in control this appeared to be more likely due to noise; the trend was much clearer in both Pch and PHZ mice.

### Parasitemia

**FIGURE 5:**
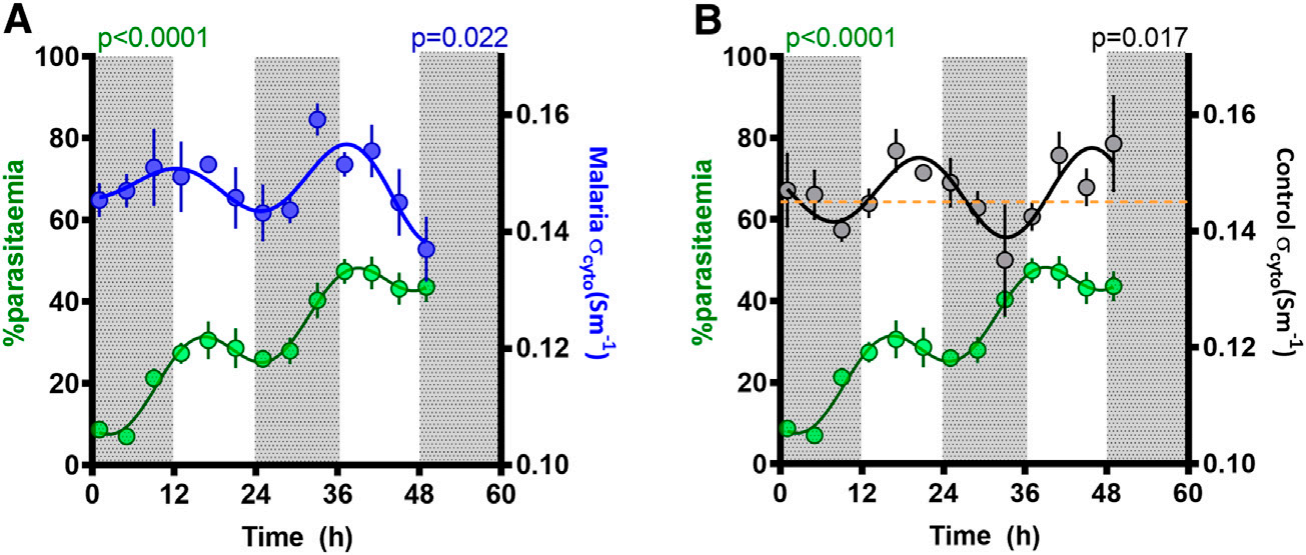
Parasitemia rhythm and correlation with cytoplasmic conductivity (σ_cyto_). Mean ± SEM and best fitting models for σ_cyto_ in **(A)** malaria (blue) and **(B)** control (black) groups, as well as parasitemia in malaria mice (green), as a function of time since the experiment began, with ZT indicated by light and dark panels. The yellow line in Figure 5B refers to the threshold of σ_cyto_ = 0.145 Sm^−1^; periods when σ_cyto_ is below this threshold coincides with times that parasitemia increases.

The proportion of RBC infected with malaria parasites increased over the 48-h sampling window, with a mean rise of 18.5% per day (Figure 5). However, superimposed on this rise, a significant (*p* < 0.0001, fitted to a cosinor relative to a straight line) rhythm in parasitemia was observed, reflecting synchronous replication, with a period of 22.5 ± 1.2 h, an amplitude of 7.3 ± 1.1%, and a peak early in the daylight phase (acrophase 15 ± 2.8 h).

## Discussion

Comparison of blood from control, anemia (PHZ), and malaria-infected (Pch) mice revealed that σ_cyto_ exhibits a circadian rhythm whose pattern differs between treatment groups. The PHZ group provides insight into effects due to anemia rather than resulting directly from parasite activities. The C_eff_ did not display any rhythmicity in either control or PHZ mice, but intriguingly, rhythmicity was observed in C_eff_ in malaria infected mice. As expected from an expanding infection with synchronous development, parasitemia was also rhythmic during each replication cycle, which is in phase with that of σ_cyto_.

We observed two cycles of the effect of malaria infection on rhythmic behavior. Rhythmicity in parasite replication generates a rise in parasitemia when parasites synchronously burst to release their progeny, and parasitemia drops as development progresses due to sequestration into tissues (to evade immune clearance). We found that parasitemia increased during the dark phase as expected (Prior et al., 2021; O’Donnell et al., 2022), and was steady or decreased during the light phase (Figure 5). Observations of concurrent electrical activity suggest two potential mechanistic links with parasite replication. Firstly, the rhythms of malarial σ_cyto_ and parasitemia were in phase across the 48 h of the experiment (Figure 5A). During this time, parasitemia increased from ∼10 to ∼50% and the onset of malarial symptoms affects both host and parasite rhythms, especially for the genotype (AJ) used here (Prior et al., 2019). Consequently, the change in phase and the observed increase in amplitude of the σ_cyto_ rhythm could be both a cause and consequence of the parasitemia rhythm. Furthermore, if σ_cyto_ (and hence cytosolic ion content) peaks in antiphase with changes in the plasma, the RBCs will experience the greater osmotic stress which could lead to RBC swelling and weakening, potentially contributing to bursting and release of progeny parasites. Further evidence for this may be found in C_eff_, which exhibited a rhythm with similar period to σ_cyto_ in this malaria infected group (in marked contrast to control cells). However, C_eff_ phase did not coincide with the malaria σ_cyto_ rhythm, showing peaks in the mid-late light period. C_eff_ is a common indicator of cell swelling, due to changes in cell radius and membrane folding. If RBCs cycle their cytosolic ion content to pre-emptively maintain osmotic balance with alterations in blood plasma osmolarity, then ceasing adaptation to this would result in swelling and shrinkage in time with the prevailing conditions. Since the control cells can be assumed to be synchronized with these environmental changes, it may suggest that Pch-infected cells are not. Interestingly, no changes were observed in measurements of the RBC radii at the overlapping time points, but the biconcave nature of RBCs means that cell swelling typically takes place in the direction orthogonal to the plane of the disc as the biconcave regions expand outwards, RBCs can expand and shrink due to osmotic pressure over a wide range with only minimal change in the disc diameter.

Roma et al. (2014), a second correlation was identified between parasitemia and σ_cyto_ in *control* cells (Figure 5B), which may suggest that this parameter could reflect RBC susceptibility to invasion. σ_cyto_ has recently been shown to be mechanistically linked to the zeta potential ζ in human RBCs (Hughes et al., 2021) and platelets (Hughes et al., 2022). ζ is the electrical potential observed outside the cell surface and determines whether the cell surface is electrically repulsive to extracellular material with a similar surface charge. Studies of the two parameters suggests that ζ hyperpolarizes when σ_cyto_ rises (and *vice versa*) and that this can alter extracellular ion concentration and antibody binding (Hughes et al., 2021; Hughes et al., 2022). A rise in the σ_cyto_ of uninfected cells, and a concomitant increase in ζ, may provide sufficient electrostatic repulsion between RBC and invading parasite to prevent contact and invasion, whilst a low σ_cyto_ and ζ may indicate greater susceptibility to invasion. It is notable that parasitemia was only observed to increase when control σ_cyto_ was below 0.145 Sm^−1^, illustrated by the broken line on Figure 5B. Whether this represents a threshold for when RBC are permissive to invasion or is a simply a coincidence of the parasite’s developmental rhythm could be tested by perturbing the temporal alignment between parasite and host rhythms and comparing invasion rates across a gradient for σ_cyto._ We suggest this aspect needs further investigation, particularly in light of the antimalarial quinine, which blocks potassium channels and may alter the electrostatic interactions between parasites and the RBCs they are attempting to invade.

Following PHZ treatment, up to 30% of the RBC in the PHZ mice are reticulocytes (Birget et al., 2017). Given that the DEP spectrum (and hence, the derived properties from that spectrum) represents an average response across the population, whilst mature RBCs are predominant, an increase in reticulocyte number may be responsible for some of these changes, rather than changes in the mature RBC population. For example, reticulocytes have a higher concentration of macromolecules than RBCs, particularly RNA. This may result in a higher intracellular water content than mature RBCs, resulting in a lower overall concentration of free ions and a lower cytoplasm conductivity. It has been reported (Moura, 2020) that human reticulocytes have a lower mean membrane potential than erythrocytes, which would also point to a lower cytoplasm conductivity (Hughes et al., 2021). As with control cells, the C_eff_ in anemic mice did not exhibit a rhythm, suggesting that RBC morphology did not change rhythmically. Given that the peaks in PHZ and control σ_cyto_ were largely similar this would suggest that cell adaptation to blood osmolarity remained similar. However, the observed C_eff_ value increased to 0.016 F/m^2^, suggesting a possible higher value of C_eff_ in the reticulocyte population due to their larger size and more convoluted morphology (Ovchynnikova et al., 2018). Reticulocytaemia of naïve mice is ∼1% and this was slightly elevated by malaria infection (but still <5%), thus RBC age structure is insufficient to explain the DEP spectrum and derived properties for the Pch group.

## Conclusion

Whilst circadian variations in RBC electrophysiology have been well-characterized under normal conditions, the role these rhythms play in, and are affected by, disease states has been largely unexplored. We have examined for the first time the electrophysiological behavior of blood (principally made of >99% of RBCs) affected by malaria and anemia over a 48 h period, and identified both similarities with, and differences from, the electrophysiological rhythms present in healthy RBCs. Analysis suggests that whilst the RBC oscillator is robust to anemia, parasite development and the systemic effects of infection may impact the RBC clock. The perturbation of the cytoplasmic conductivity rhythm could be both a cause and consequence of rhythmic parasite replication, potentially affecting when RBC burst to initiate the next cycle of replication and the ease by which progeny parasites can invade RBC. Determining how electrophysiological oscillations of RBC impact on parasite activities and *vice versa* might reveal new ways to target parasites and to lessen the severity of disease symptoms.

## Data Availability

The raw data supporting the conclusions of this article will be made available by the authors, without undue reservation.
